# Health-related quality of life outcomes among vedolizumab-treated patients with inflammatory bowel disease in the UK and Ireland: a 52-week observational study (OCTAVO)

**DOI:** 10.1186/s41687-025-00846-9

**Published:** 2025-07-01

**Authors:** Gareth Parkes, Ayesha Akbar, Ian Beales, Martin Buckley, Tom Creed, Said Din, Nikolas Plevris, Niamh Hogan, Nicola Heggs, Simon Meadowcroft, Mike Wallington, Aileen Fraser

**Affiliations:** 1 Gastroenterology, The Royal London Hospital, London, E1 1FR UK; 2 Gastroenterology, St Mark’s Hospital, London, HA1 3UJ UK; 3 Gastroenterology, Norfolk and Norwich University Hospital, Norwich, NR4 7UY UK; 4 Gastroenterology, Mercy University Hospital, Cork, T12 WE28 Ireland; 5 Gastroenterology, Bristol Royal Infirmary, Bristol, BS2 8HW UK; 6 Gastroenterology, Royal Derby Hospital, Derby, DE22 3NE UK; 7 Gastroenterology, Western General Hospital, Edinburgh, EH4 2XU UK; 8 Takeda UK, London, W2 6BD UK; 9 Department of Biostatistics, OPEN VIE, Marlow, SL7 2FF UK

**Keywords:** Crohn’s disease, Gastroenterology, Observational study, Quality of life, Real-world study, Ulcerative colitis, Vedolizumab

## Abstract

**Background:**

Vedolizumab is a gut-selective, anti–lymphocyte trafficking biologic therapy for inflammatory bowel disease (IBD). Clinical trials have demonstrated the positive impact of vedolizumab on patient quality of life (QoL); however, real-world evidence of its impact is limited. We evaluated vedolizumab impact on QoL of patients with IBD during the first 52 weeks of treatment in real-world practice in the United Kingdom and Ireland.

**Methods:**

In this prospective observational study, patients with IBD initiated on vedolizumab completed 4 validated IBD-specific QoL questionnaires at baseline and weeks 14, 26, and 52. The primary endpoint was change in mean Short Inflammatory Bowel Disease Questionnaire (SIBDQ) score. Secondary endpoints included changes in mean scores and sub-scores for other QoL questionnaires.

**Results:**

Overall, 61 patients were enrolled, including 22 with Crohn’s disease (CD) and 39 with ulcerative colitis (UC). At week 52, the mean change in SIBDQ scores from baseline was statistically significant (+ 12.3 [*p* = 0.0123] for CD and + 10.8 [*p* = 0.0037] for UC) and clinically meaningful (considered as a ≥ 10-point change). A significant improvement in mean SIBDQ scores was seen as early as week 14 for both CD and UC cohorts (*p* = 0.0256 and *p* = 0.0348, respectively).

**Conclusions:**

These real-world findings, using multiple validated tools, demonstrate that vedolizumab treatment for IBD is associated with measurable improvements in QoL from baseline.

**Supplementary Information:**

The online version contains supplementary material available at 10.1186/s41687-025-00846-9.

## Background

Inflammatory bowel disease is a condition caused by chronic inflammation of the digestive tract and is characterized by symptoms of bloody diarrhea and/or abdominal pain. Inflammatory bowel disease is an umbrella term that covers 2 distinct diseases: Crohn’s disease (CD) and ulcerative colitis (UC) [1]. Due to the nature of disease-related symptoms and associated treatment, CD and UC can severely affect patient emotional health, social and leisure activities, work productivity, and employment [2–6]. Patients with increased disease activity typically have a lower QoL, whereas patients in remission have a QoL similar to that of the general population [4, 7]. Improvement in health-related quality of life (HRQoL) is an important treatment goal for patients [8].

Both CD and UC are characterized by the ongoing recruitment of leukocytes to the gut, which leads to amplification of the inflammatory response, ultimately resulting in tissue damage within the affected areas [1]. Treatment for CD and UC depends on disease severity and guidelines recommend conventional therapy such as steroids and/or thiopurines for both CD and UC, and 5-aminosalicylates specifically for UC, as well as biologics such as tumor necrosis factor inhibitors (infliximab, adalimumab), the interleukin 12/23-receptor antagonist-ustekinumab, or the anti-integrin antibody-vedolizumab [9, 10]. Vedolizumab is a gut-selective, anti–lymphocyte trafficking agent that reduces mucosal inflammation, decreases T-cell migration to the gastrointestinal mucosa [11], and has demonstrated clinical efficacy in CD [12] and UC [13, 14]. While vedolizumab has also shown positive effects on patient HRQoL in clinical trials [15, 16], there is a dearth of long-term, prospective studies that use multiple HRQoL measures.

The OCTAVO study is a multicohort, multicenter study investigating the real-world use of vedolizumab in the United Kingdom (UK) and Ireland. The current study aimed to understand the effect of vedolizumab on HRQoL in a cohort of patients with UC or CD across a number of dimensions, including disease control, disease symptoms, emotional health, social function, work productivity, body stigma, and sexual intimacy.

## Methods

### Study aim

The aim of the current study (European post-authorization study number: EUPAS22954) was to evaluate the impact of vedolizumab therapy on the QoL of patients with CD and UC during the first 52 weeks following treatment initiation.

### Study design and data source

Patients who had initiated treatment with intravenous vedolizumab for CD or UC were identified from the following 7 participating centers across the UK and Ireland: The Royal London Hospital, England; St Mark’s Hospital, England; Bristol Royal Infirmary, England; Royal Derby Hospital, England; Western General Hospital, Scotland; Norfolk and Norwich University Hospital, England; and Mercy University Hospital, Ireland. The centers were chosen for geographical spread and to maximize patient recruitment.

QoL data were collected prospectively during 52 weeks of treatment with vedolizumab. Data were collected from all patients who met the eligibility criteria (below) and who gave consent. The data collection window lasted for 23 months between July 31, 2018, and June 30, 2020. Patient-reported outcomes data, including HRQoL data, and data on patient experiences and preferences when choosing inflammatory bowel disease treatment (at baseline only) were collected directly from patients through a bespoke online survey developed in association with a third party (First Line Research) [17]. The online questionnaire was hosted by Confirmit [18] on their secure servers [19]. OPEN VIE Ltd (Marlow, United Kingdom) could access online progress reports to confirm recruitment and questionnaire completion through a dedicated portal.

QoL was evaluated using 4 established and validated questionnaires that are designed to evaluate the experience of patients with inflammatory bowel disease. The questionnaires were chosen to cover a wide range of aspects of patient QoL in the scores they report, including disease control, disease symptoms, emotional health, social function, body stigma, sexual intimacy, and work productivity, and are described below.

The Short Inflammatory Bowel Disease Questionnaire (SIBDQ) [5] comprises 10 items covering 4 domains (bowel symptoms, systemic symptoms, emotional health, and social function), and each item is scored on a 7-point Likert scale, with higher scores indicating better functioning. A 10-point or greater change in overall SIBDQ score was considered clinically meaningful in this study.

The Inflammatory Bowel Disease-Control-8 questionnaire (IBD-C-8) [20] was designed to measure overall disease control from the patient perspective. The questionnaire consists of 8 questions, each scored between 0 and 2. No clinically meaningful threshold is established for the IBD-C-8; higher scores indicate better perceived inflammatory bowel disease control.

The Work Productivity and Activity Impairment questionnaires specific to CD and UC (WPAI-CD/UC) [21–23] are 6-item surveys that measure the work-related impact of inflammatory bowel disease over the past 7 days in terms of absenteeism, presenteeism, overall work impairment, and impairment of nonwork activities. Results are expressed as a percentage, with higher percentages indicating greater impairment. A change in score of 7% is considered clinically meaningful [23]. Patients were asked whether they were employed (working for pay) at baseline and weeks 14, 26, and 52, and percentages were calculated with the total number of employed patients at each time point as the denominator.

The Rating Form of Inflammatory Bowel Disease Patient Concerns (RFIPC) [6] comprises 25 questions covering 4 domains (disease impact, disease complications, sexual intimacy, and body stigma) scored on a 0–100 visual analog scale. No clinically meaningful threshold is established for the RFIPC; higher scores indicate higher disease-related concerns.

All 4 questionnaires were answered at baseline, week 14, week 26, and week 52. A bespoke questionnaire evaluating patient involvement in the treatment decision–making process was also answered at baseline. All 5 questionnaires can be found in full in the Supplementary Information.

A favorable ethical opinion was gained from the East Midlands–Nottingham 2 Research Ethics Committee (REC) on May 14, 2018 (REC reference: 18/EM/0094) for the conduct of the study in England. For Ireland, a favorable ethical opinion was granted by the Clinical REC of the Cork Teaching Hospitals, University College Cork, on January 18, 2018 (reference: ECM 3 (r) 10/01/18). Approval from the Research and Development department of each institution was obtained before study commencement.

### Patients

Data were collected from patients with a diagnosis of UC or CD who initiated treatment with intravenous vedolizumab as an outpatient for the first time at study enrollment and were aged over 18 years. Patients with acute severe disease with primary fistulizing disease and patients enrolled in an interventional clinical trial were excluded. All patients provided consent to participate in the study before vedolizumab initiation.

### Endpoints

The primary endpoint was a change in mean SIBDQ score from baseline to week 14, week 26, and week 52. Secondary endpoints included changes in mean scores and sub-scores for IBD-C-8, RFIPC, and WPAI-CD/UC from baseline to week 14, week 26, and week 52 and description of patient experiences and preferences when choosing inflammatory bowel disease treatment at baseline only.

### Data analysis

Data from all centers were pooled, and patients were separated into CD and UC cohorts for data analysis. The mean and standard deviation (SD) for overall questionnaire scores (and for sub-scores, where relevant) were calculated using all data available from patients who were receiving vedolizumab at each time point. Patients who had discontinued vedolizumab were excluded from analyses following the date of discontinuation. Where data were missing, they were dealt with according to instrument guidelines where available [20, 24]. If no guidelines were available, data were analyzed using available data only, with no imputation for missing data. Percentages were calculated with the total number of patients in the respective cohort as the denominator.

The Mann-Whitney test was used to evaluate statistical significance of the change in mean SIBDQ overall score between baseline and week 14, baseline and week 26, and baseline and week 52. p-values < 0.05 were considered statistically significant.

A repeated-measures ANOVA test was used to determine whether there were any statistically significant effects of time on changes in the questionnaire scores during the 12 months post initiation of vedolizumab. Where a violation of Mauchly’s test of sphericity was observed, appropriate corrections were applied (e.g., for epsilon > 0.75, the Huynh-Feldt correction was applied, and for epsilon < 0.75, the Greenhouse-Geisser correction was applied). A sample size of 30 patients each with CD and UC was chosen; these size cohorts were calculated to have 75% power to detect a statistically significant (*p* < 0.05) and clinically meaningful (≥ 10-point) change in SIBDQ score at week 52. Statistical analyses were performed using Stata 14.2 (StataCorp LLC) and Microsoft Excel.

## Results

### Patients

Sixty-nine patients from the 7 participating centers met the eligibility criteria and gave consent to be included. Eight patients were excluded from the analysis (6 did not complete the baseline assessment and 2 did not pass the screening criteria). In total, 61 patients participated in the study: 22 patients with CD and 39 with UC.

Baseline characteristics are shown in Table 1.

**Table 1:** Baseline characteristics of patients enrolled

	Characteristic	Crohn’s disease (*n* = 22)	Ulcerative colitis (*n* = 39)
Sex	Female	13 (59%)	17 (44%)
Age at index (years)	18 to < 30	2 (9%)	9 (23%)
	30 to < 50	14 (64%)	16 (41%)
	50 to < 70	3 (14%)	10 (26%)
	≥ 70	3 (14%)	4 (10%)
	Median (IQR)	40.5 (34.8–52.0)	39.0 (30.0–56.0)
Age at diagnosis (years)	Median (IQR)	28.0 (20.8–37.3)	29.0 (22.0–39.0)
Duration of disease at treatment initiation (years)	Median (IQR)	11.5 (5.3–17.5)	5.6 (1.3–17.4)

IQR, interquartile range

### Questionnaire responses

The number of patients who were receiving vedolizumab and completed the 4 QoL measures at each time point are shown in Table 2. At baseline, all patients with CD (22, 100%) and 38/39 (97%) patients with UC provided responses to all of the questionnaires. At week 52, 93% (*n* = 14/15) of patients with CD completed the 4 questionnaires and 85% (*n* = 29/34) of patients with UC completed the SIBDQ, IBD-C-8, and RFIPC questionnaires. Responses for the WPAI-CD/UC questionnaires related to absenteeism, presenteeism, and work impairment were provided by patients who were employed.

**Table 2:** Number and percentage of patients who responded to quality-of-life questionnaires at each time point

	Crohn’s disease				Ulcerative colitis			
	Baseline	Week 14	Week 26	Week 52	Baseline	Week 14	Week 26	Week 52
**Number of patients receiving vedolizumab**,** N**	22	21	20	15	39	38	36	34
**Number of questionnaire responses from patients receiving vedolizumab**,** n (%**^**a**^)								
SIBDQ responses	22 (100%)	18 (86%)	18 (90%)	14 (93%)	38 (97%)	33 (87%)	32 (89%)	29 (85%)
IBD-C-8 responses	22 (100%)	18 (86%)	18 (90%)	14 (93%)	38 (97%)	33 (87%)	32 (89%)	29 (85%)
RFIPC responses	22 (100%)	18 (86%)	18 (90%)	14 (93%)	38 (97%)	33 (87%)	32 (89%)	29 (85%)
WPAI nonwork activity impairment responses	22 (100%)	18 (86%)	18 (90%)	14 (93%)	38 (97%)	33 (87%)	32 (89%)	28 (82%)
**Number of employed patients receiving vedolizumab**,** N**	12	8	8	6	24	18	19	17
**Number of questionnaire responses from patients receiving vedolizumab**,** n (%**^**b**^**)**								
WPAI absenteeism responses	11 (92%)	7 (88%)	7 (88%)	5 (83%)	23 (96%)	17 (94%)	19 (100%)	17 (100%)
WPAI presenteeism responses	10 (83%)	7 (88%)	6 (75%)	5 (83%)	22 (92%)	16 (89%)	18 (95%)	17 (100%)
WPAI work impairment responses	10 (83%)	7 (88%)	6 (75%)	5 (83%)	22 (92%)	16 (89%)	18 (95%)	17 (100%)

^a^Percentages measured as a percentage of all patients receiving vedolizumab at that time point

^b^For employment-related questionnaires, percentages are measured as a percentage of all employed patients receiving vedolizumab at that time point

IBD-C-8, Inflammatory Bowel Disease-Control-8 questionnaire; RFIPC, Rating Form of Inflammatory Bowel Disease Patient Concerns; SIBDQ, Short Inflammatory Bowel Disease Questionnaire; WPAI, Work Productivity and Activity Impairment questionnaire.

### QoL questionnaire results

**Fig. 1:**
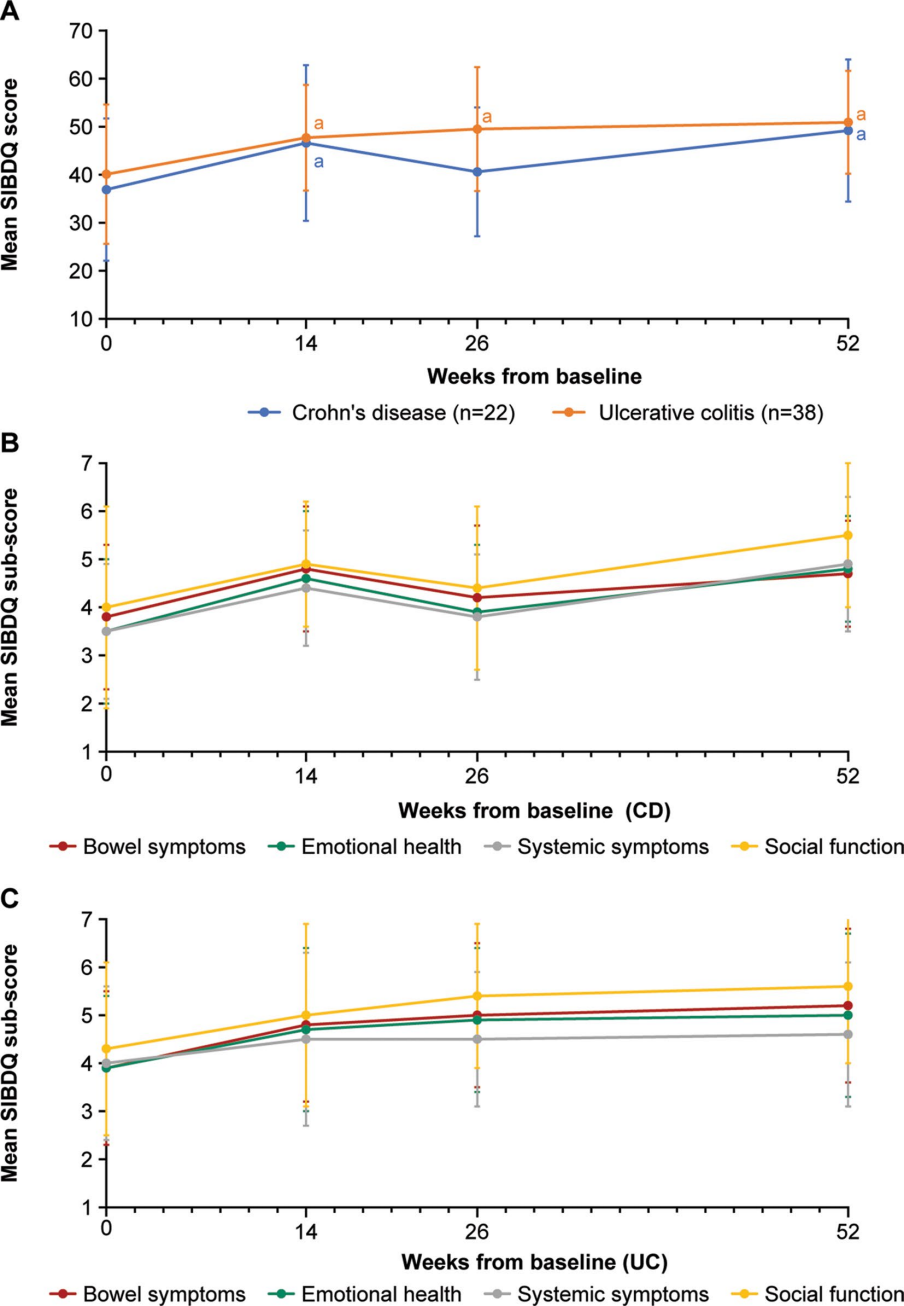
SIBDQ mean scores in patients over 52 weeks of treatment with vedolizumab. Mean overall scores (**A**) and sub-scores in patients with Crohn’s disease (**B**) and with ulcerative colitis (**C**) among patients receiving vedolizumab. Higher scores = better functioning. Minimum score = 10 (indicating “poor health-related quality of life”), maximum score = 70 (indicating “optimum health-related quality of life”) for overall scores in Fig. 1A. Minimum score = 1 (indicating “all of the time”), maximum score = 7 (indicating “none of the time”) for all sub-scores. Points indicate mean scores for all patients on treatment and completing the questionnaire; error bars indicate standard deviation. A ≥ 10-point improvement was considered clinically meaningful for the overall SIBDQ score. ^a^Indicates *p* < 0.05 for comparison with baseline (only calculated for overall score, not for sub-scores). CD, Crohn’s disease; SIBDQ, Short Inflammatory Bowel Disease Questionnaire; UC, Ulcerative colitis

A statistically significant and clinically meaningful (≥ 10-point increase) improvement in mean SIBDQ score at week 52 (versus baseline) was seen for both patients with CD (difference in mean scores 12.3; *p* = 0.0123) and UC (difference in mean scores 10.8; *p* = 0.0037; Fig. 1A) who remained on vedolizumab for the whole observation period. Furthermore, a statistically significant improvement in SIBDQ scores compared with baseline was seen at the first time point following baseline (week 14) for patients in both cohorts (CD *p* = 0.0256, UC *p* = 0.0348); at this time point, 56% (*n* = 10/18) of patients with CD and 27% (*n* = 9/33) with UC had clinically meaningful improvement in SIBDQ score. While among patients with CD, there was a subsequent fall in the mean SIBDQ score at week 26, with clinically meaningful improvements observed in 22% (*n* = 4/18) of patients, among those with UC, the mean SIBDQ scores followed an upward trajectory, with 31% (*n* = 10/32) of patients showing clinically meaningful improvement. Clinically meaningful improvements were seen in 50% (*n* = 7/14) of patients with CD and 38% (*n* = 11/29) with UC at week 52. Analysis of the effect of time on the change in overall SIBDQ score showed an overall improvement in scores from baseline over the 52 weeks post index for patients with CD (*p* = 0.018, *n* = 14) and UC (*p* < 0.001, *n* = 29).

Changes in mean SIBDQ sub-scores are shown in Fig. 1B and C and Table S1. Similar patterns of improvements in QoL were seen for all sub-scores, including emotional health, social function, and systemic symptoms.

**Fig. 2:**
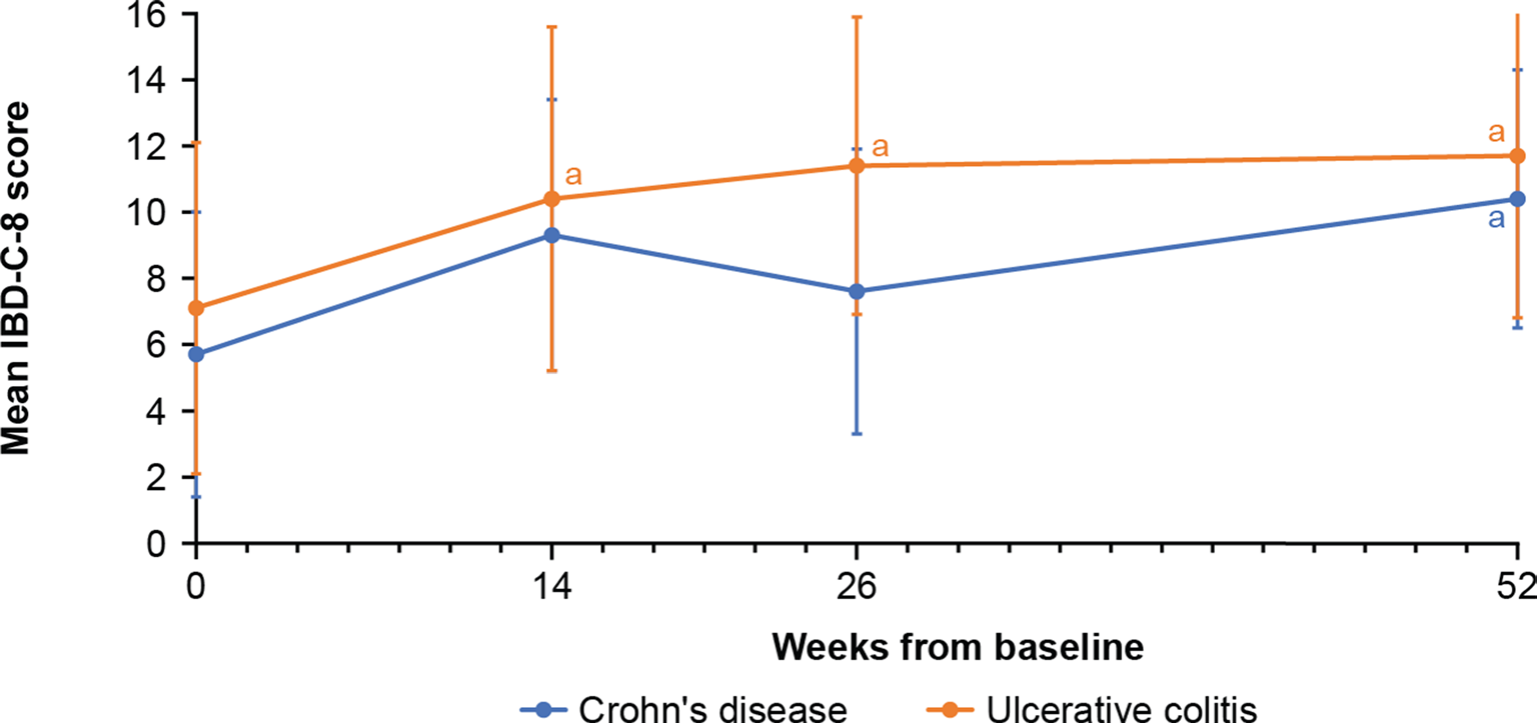
IBD-C-8 mean scores among patients receiving vedolizumab. Higher scores = better perceived disease control. Minimum score = 0, maximum score = 16. Points indicate mean scores for all patients on treatment and completing the questionnaire; error bars indicate standard deviation. ^a^Indicates *p* < 0.05 for comparison with baseline. IBD-C-8, Inflammatory Bowel Disease-Control-8 questionnaire

Change in mean IBD-C-8 scores over the treatment period can be seen in Fig. 2. The mean (SD) overall IBD-C-8 score for patients with CD was 5.7 (4.3) at baseline (*n* = 22), 9.3 (4.1) at week 14 (*n* = 18), 7.6 (4.3) at week 26 (*n* = 18), and 10.4 (3.9) at week 52 (*n* = 14). For patients with UC, the mean (SD) overall IBD-C-8 score was 7.1 (5.0) at baseline (*n* = 38), 10.4 (5.2) at week 14 (*n* = 33), 11.4 (4.5) at week 26 (*n* = 32), and 11.7 (4.9) at week 52 (*n* = 29). There was statistically significant effect of time on the change in the total IBD-C-8 score from baseline over the 52 weeks post-index for patients with CD (*p* = 0.010, *n* = 14) and UC (*p* < 0.001, *n* = 14). In line with the results seen for the SIBDQ overall score, there was a trend for IBD-C-8 scores to improve as early as week 14, and the improvements were sustained until week 52 among patients who remained on vedolizumab.

**Fig. 3:**
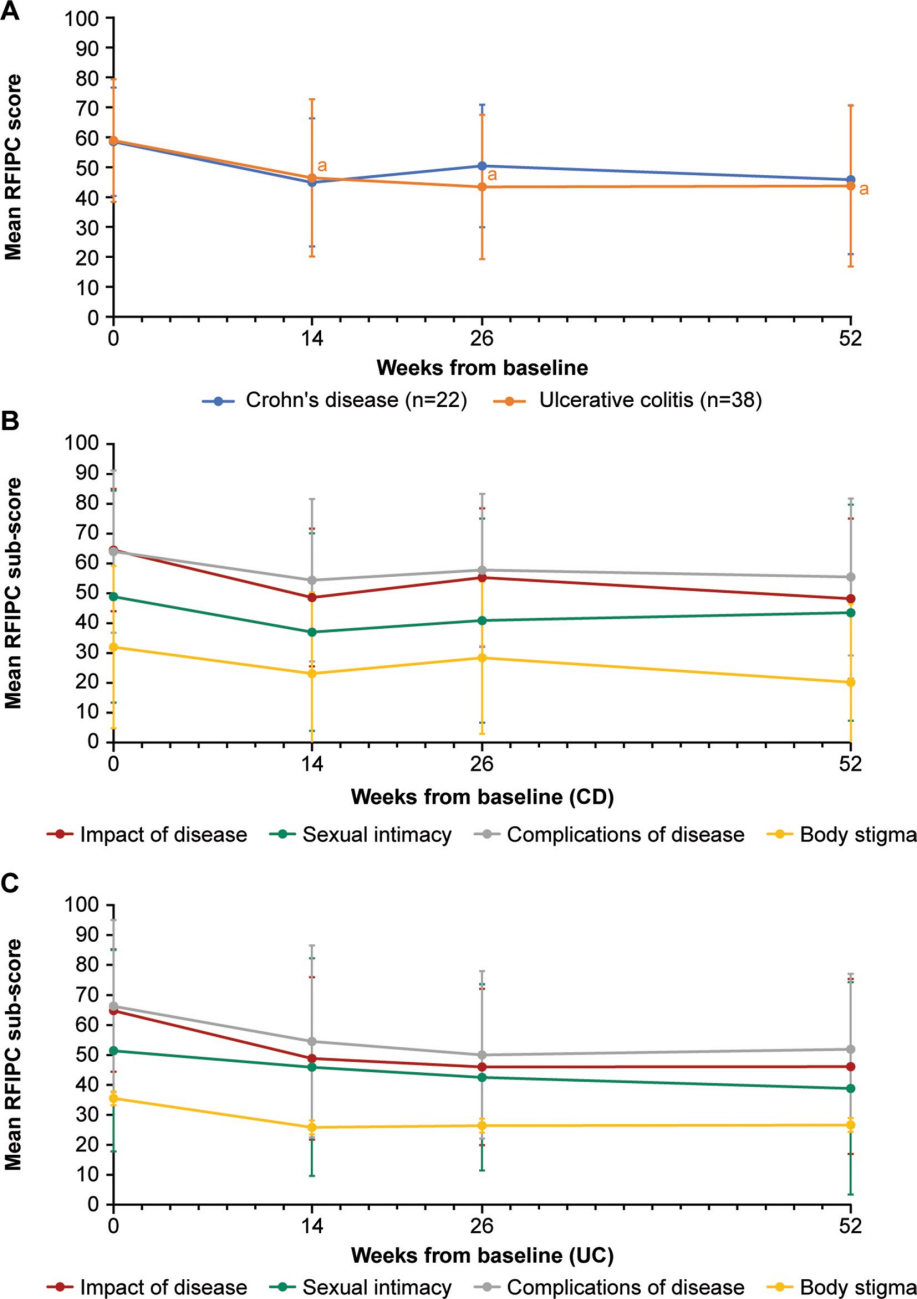
RFIPC mean scores in patients over 52 weeks of treatment with vedolizumab. Mean overall scores (**A**) and sub-scores for patients with Crohn’s disease (**B**) and with ulcerative colitis (**C**) among patients receiving vedolizumab. Lower scores = lower concern. Minimum score = 0, maximum score = 100. Points indicate mean scores for all patients on treatment and completing the questionnaire; error bars indicate standard deviation. ^a^Indicates *p*‍<‍0.05 for comparison with baseline (only calculated for overall score, not for sub-scores). CD, Crohn’s disease; RFIPC, Rating Form of Inflammatory Bowel Disease Patient Concerns; UC, Ulcerative colitis

The change in RFIPC mean overall scores and sub-scores over the treatment period can be seen in Fig. 3 and Table S2. There was a trend for overall scores and all sub-scores (including body stigma and sexual intimacy sub-scores) to reduce, consistent with the improvement in status over the 52-week treatment period and in line with the results from the SIBDQ and IBD-C-8 scores. There was a mean (SD) reduction in overall score from baseline of 11.5 (20.3) points at week 14 (*n* = 33), 14.1 (20.1) points at week 26 (*n* = 32), and 14.7 (20.0) points at week 52 for UC (*n* = 29). For patients with UC, there was a statistically significant effect of time on the change in overall RFIPC score, signifying an overall improvement in scores from baseline to week 52 post-index (*p* < 0.001, *n* = 29). While there was a general trend suggesting that overall RFIPC scores improved post-index, the effect of time on the change in overall RFIPC score among patients in the CD cohort post-index did not reach statistical significance (*p* = 0.055, *n* = 14). The improvement could be seen as early as week 14 in both cohorts.

**Fig. 4:**
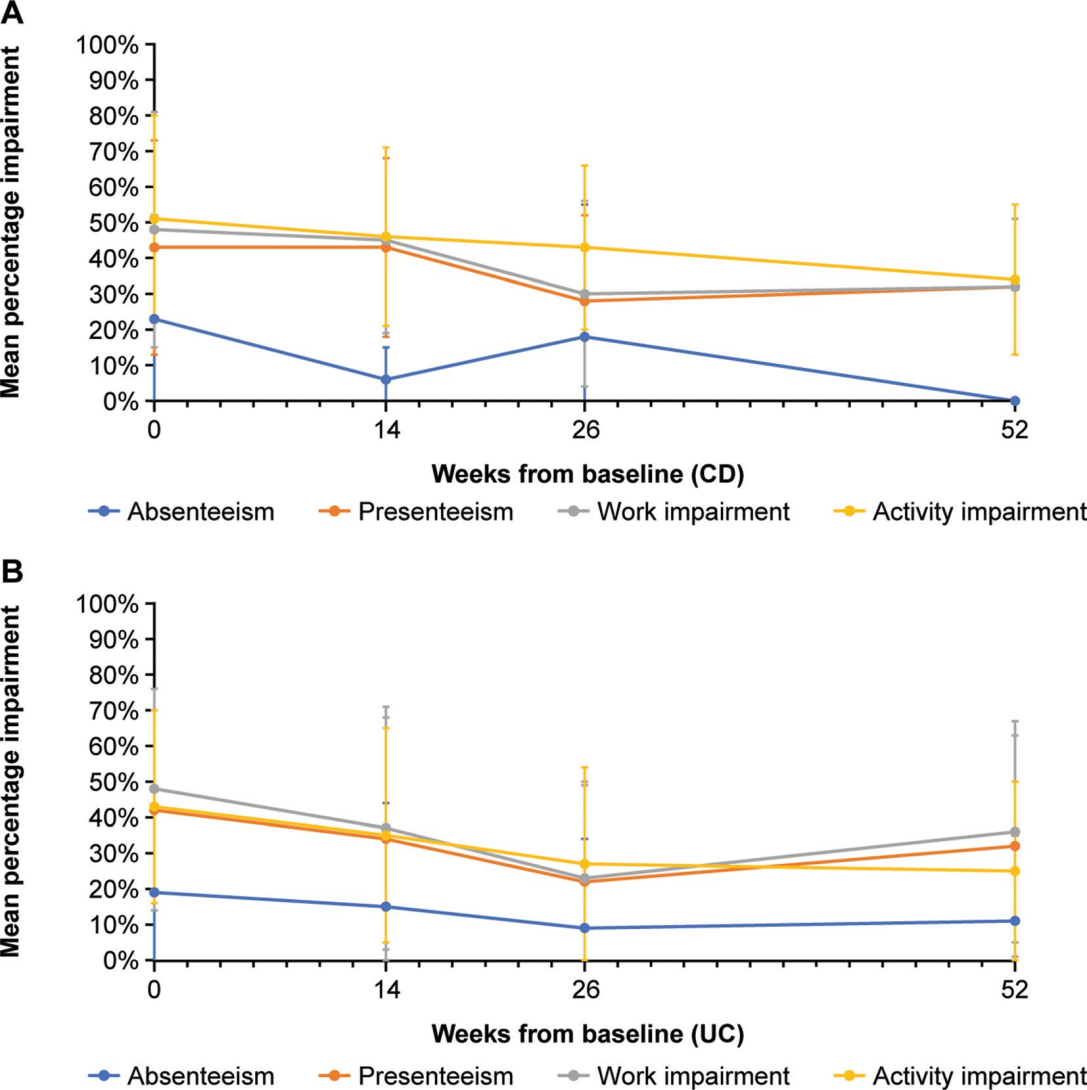
WPAI-CD/UC mean scores in patients over 52 weeks of treatment with vedolizumab. Mean subdomain scores over treatment period for patients with Crohn’s disease^a^ (**A**) and ulcerative colitis (**B**) treated with vedolizumab. Lower scores = lower impairment. Points indicate mean scores for all patients on treatment and completing the questionnaire (and in employment, where required by the questionnaire); error bars indicate standard deviation. ^a^Five patients with CD who were employed and were receiving vedolizumab at week 52 answered the WPAI-CD at week 52. All patients reported no time off work (absenteeism) related to the disease. CD, Crohn’s disease; WPAI-CD, Work Productivity and Activity Impairment questionnaire specific to Crohn’s disease; UC, Ulcerative colitis

Changes in the 4 dimensions of WPAI-CD/UC (3 questions related to work productivity and 1 question related to nonwork activity) can be seen in Fig. 4. As with previous results, there was a trend toward reduction in the WPAI-CD/UC score (indicative of improved status), which was visible as soon as week 14 and was generally sustained through week 52 for both CD and UC cohorts. At week 52, this improvement versus baseline was clinically meaningful (≥ 7% difference in mean scores [23]) for all sub-scores in both CD and UC cohorts (except for the presenteeism sub-score in the UC cohort, where there was a 6% reduction). A statistically significant effect of time on changes in the WPAI-CD/UC mean scores was observed for presenteeism, work impairment, and activity impairment in patients with UC.

At baseline, all patients completed a bespoke questionnaire that assessed their involvement in decisions about their treatment. When presented with the statement “I want to be involved in choosing my treatment,” 86% (*n* = 19/22) of patients with CD and 87% (*n* = 34/39) with UC either “somewhat” or “totally agreed.” Additionally, 95% (*n* = 21/22) of patients with CD and 72% (*n* = 28/39) with UC “agreed” or “totally agreed” with the statement “I was involved in choosing my treatment.” However, only 74% (*n* = 14/19) of patients with CD and 65% (*n* = 24/37) of patients with UC “somewhat agreed” or “totally agreed” with the statement “I received enough information on different treatment options to help me to choose the best treatment for me.”

## Discussion

Patient-reported outcomes are increasingly used to monitor disease activity both in clinical practice and as endpoints in clinical trials [25]. Literature, including the STRIDE II study, suggests that improving QoL is an important treatment goal both for patients with inflammatory bowel disease and to reduce societal costs from the disease [26, 27]. This study examined the impact of vedolizumab treatment on select, validated patient-reported outcome measures chosen to cover a wide range of QoL domains including overall QoL and work productivity.

Over 52 weeks of treatment, patients receiving vedolizumab for CD or UC showed improvements in a range of validated patient health–related QoL instruments. For the primary endpoint (change in mean overall SIBDQ score over the 52-week treatment period), statistically significant improvements were seen as early as week 14 in both patient cohorts and were maintained until week 52. For both cohorts at week 52, the increase in mean SIBDQ score from baseline was above the threshold for a clinically meaningful improvement (≥ 10-point) used in this study. SIBDQ score is correlated with disease activity, such that patients with active disease report significantly lower SIBDQ scores compared with those in remission [28]. Our findings of improved SIBDQ scores from baseline thus signify the clinical relevance of these results. Considering that vedolizumab is a gut-selective therapy, it is noteworthy that patients in both cohorts reported similar improvements in both bowel and systemic symptoms (as indicated by increase in the SIBDQ sub-scores) at weeks 14 and 52.

A similar trend was observed for IBD-C-8 scores; this measure was chosen as it has been shown to be a rapid, reliable, valid, and sensitive instrument for measuring overall disease control from the patient perspective [20]. Results from the overall RFIPC scores also showed a similar trend, and sub-scores for this measure suggested a trend toward improvement in measures of sexual intimacy and body stigma. Both these measures are important concerns for patients and are not directly captured in other measures of patient QoL. Furthermore, there was a consistent trend of improvement in measures of work productivity and nonwork activity from the WPAI-CD/UC. Again, these improvements were visible by week 14 in both cohorts and were generally sustained until week 52. Given that the majority of participants in our study were of working age and that inflammatory bowel disease has been shown to significantly impact the quality of working life [29], it is of interest that vedolizumab may help to relieve aspects of work- and activity-related difficulties in some patients with IBD.

As expected, results of the bespoke questionnaire suggested that most patients want to be (86–87%), and very frequently are (72–95%), involved in decisions about their treatment for inflammatory bowel disease. However, fewer patients (65–74%) reported receiving enough information about treatment options than patients who reported wanting to or being involved in the decision. These results represent an improvement on a study conducted in 2007, which found that 39% of patients had been spoken to by their doctor about new treatments [3]. Our data suggest that improvement is still necessary, given that the percentage of patients who were given enough information about treatments is lower than the percentage that wanted to be involved in treatment decisions.

This study has some limitations. The dataset analyzed is small (22 patients with CD and 39 with UC) and exacerbated by the loss to follow-up, and thus conclusions may have limited applicability to the wider population. The limited patient population was particularly acute when considering responses to the WPAI-CD/UC. Some aspects of this questionnaire could only be completed by patients in employment, resulting in smaller cohorts than those for the other questionnaires. After baseline, < 20 patients with UC and < 10 patients with CD responded to the WPAI-CD/UC sections about employment.

Since only those patients who remained on vedolizumab were included in the analyses, the QoL of patients who discontinued treatment within 12 months of being initiated on vedolizumab is unclear. Treatment discontinuations may also have had an effect on patterns observed in overall QoL questionnaire scores. For instance, an unusual trend is seen in the SIBDQ overall scores and sub-scores, IBD-C-8 overall scores, and RFIPC overall scores and sub-scores for patients with CD: there appears to be a worsening in QoL between weeks 14 and 26 and a subsequent improvement between weeks 26 and 52. A possible explanation lies with the number of treatment discontinuations in this period. Of 22 patients with CD who initiated vedolizumab treatment, 5 discontinued treatment between weeks 26 and 52 (out of a total of 7 discontinuations in all 52 weeks for the CD cohort). It is possible that these 5 patients were experiencing reduced therapeutic benefit with vedolizumab and therefore discontinued treatment, with the caveat that efficacy was not assessed in the current study. With these 5 patients no longer being counted for mean scores at week 52, this may explain the subsequent improvement in mean SIBDQ, IBD-C-8, and RFIPC scores seen between weeks 26 and 52. Additionally, there may be many factors that can influence therapeutic efficacy and/or patient-reported outcomes in patients with IBD such as medication adherence or patient age, sex, disease activity at baseline, and prior surgeries [30, 31]. These were not evaluated in the study.

Finally, this was a single-arm study in which all patients received vedolizumab. While we observed a general increase in patient QoL from the start of treatment, we cannot directly compare this with results from another therapy, or with no treatment, over the same time period.

## Conclusions

This study shows that real-world treatment with vedolizumab for inflammatory bowel disease is associated with a measurable, clinically relevant improvement in QoL, as assessed using 4 distinct, validated QoL tools. The improvement in QoL was seen across a range of domains, including symptom control, emotional health, social function, work productivity, body stigma, and sexual intimacy. An improvement in these measures was observed as soon as week 14 and was generally sustained throughout the year-long course of treatment.

## Electronic supplementary material

Below is the link to the electronic supplementary material.

Supplementary Material 1

Supplementary Material 2

## Data Availability

The data that support the findings of this study are not openly available due to reasons of sensitivity and are available from the corresponding author upon reasonable request. Data are located in controlled access data storage at servers belonging to Takeda UK.

## Abbreviations

CD: Crohn’s disease
HRQoL: Health-related quality of life
IBD: Inflammatory bowel disease
IBD-C-8: Inflammatory Bowel Disease-Control-8 questionnaire
QoL: Quality of life
REC: Research Ethics Committee
RFIPC: Rating Form of Inflammatory Bowel Disease Patient Concerns
SD: Standard deviation
SIBDQ: Short Inflammatory Bowel Disease Questionnaire
UC: Ulcerative colitis
UK: United Kingdom
WPAI-CD: Work Productivity and Activity Impairment questionnaire specific to Crohn’s disease
WPAI-CU: Work Productivity and Activity Impairment questionnaire specific to ulcerative colitis
