# Supplementary material for: Health-related quality of life outcomes among vedolizumab-treated patients with inflammatory bowel disease in the UK and Ireland: a 52-week observational study (OCTAVO)
Source: J Patient Rep Outcomes. 2025 Jul 1;9:80. doi: 10.1186/s41687-025-00846-9 (PMC12214154; doi:10.1186/s41687-025-00846-9)
Supplement: Supplementary file 1 — Supplementary Material 1 [file 41687_2025_846_MOESM1_ESM.pdf]

Supplementary information

Health-related quality of life outcomes among vedolizumab-treated patients with inflammatory bowel disease in the UK and Ireland: a 52-week observational study (OCTAVO)

A. The Inflammatory Bowel Disease Control questionnaire

B. The Rating Form of Inflammatory Bowel Disease Patient Concerns questionnaire

C. Short Quality of Life Questionnaire for Inflammatory Bowel Disease

D. The Work Productivity and Activity Impairment Crohn's disease questionnaire

E. The Work Productivity and Activity Impairment ulcerative colitis questionnaire

F. OCTAVO-Patient experience questionnaire for baseline data collection

IBD-Control

Inflammatory Bowel Disease Control Questionnaire

**1** **Do you believe that:**	Yes	No	Not sure
**a.** Your IBD has been well controlled in the past *two weeks*?	☐	☐	☐
**b.** Your *current treatment* is useful in controlling your IBD?	☐	☐	☐

(if you are not taking any treatment, please tick this box ☐ *)*

**2** **Over the past 2 weeks, have your bowel symptoms been getting worse, getting better or not changed?**	Better	No change	Worse
	☐	☐	☐

**3** **In the past 2 weeks, did you:**	Yes	No	Not sure
**a.** Miss any planned activities because of IBD? *(e.g. attending school/college, going to work or a social event)*	☐	☐	☐
**b.** Wake up at night because of symptoms of IBD?	☐	☐	☐
**c.** Suffer from significant pain or discomfort?	☐	☐	☐
**d.** Often feel lacking in energy (fatigued)? *(by 'often' we mean more than half of the time)*	☐	☐	☐
**e.** Feel anxious or depressed because of your IBD?	☐	☐	☐
**f.** Think you needed a change to your treatment?	☐	☐	☐

**4** **At your next clinic visit, would you like to discuss:**	Yes	No	Not sure

a. Alternative types of drug for controlling IBD? ☐ ☐ ☐

b. Ways to adjust your own treatment? ☐ ☐ ☐

c. Side effects or difficulties with using your medicines? ☐ ☐ ☐

d. New symptoms that have developed since your last visit? ☐ ☐ ☐

5 How would you rate the OVERALL control of your IBD in the past *two weeks*?

Please draw a vertical line (|) on the scale below

Worst possible control ____ Best possible control

The development of the IBD-Control questionnaire by the University of Liverpool was supported by an unrestricted research grant from AbbVie.

Job code: AXHUG131379(1)a
Date of preparation: August 2015

The Rating Form of Inflammatory Bowel Disease Patient Concerns questionnaire

SECTION 2

YOUR CONCERNS RELATED TO YOUR ILLNESS

Today's Date

_____/_____/_____

M M D D Y Y

Patients often develop concerns or worries related to their disease. Please place a vertical mark (I) anywhere across the line between "Not at all" and "A great deal" that indicates how you see yourself. Please respond to every statement according to how you feel today.

EXAMPLE:

Because of your condition, how concerned are you with...

SYMPTOM FLARE-UPS

Not at all ________|____________________ A great deal

I have marked the line to indicate that I feel mildly concerned. (If you feel more or less concern you would place your mark closer to the end which describes your level of concern.)

BECAUSE OF YOUR CONDITION, HOW CONCERNED ARE YOU WITH...

1. FINANCIAL DIFFICULTIES

Not at all ____________________________ A great deal

2. PAIN OR SUFFERING

Not at all ____________________________ A great deal

3. YOUR ABILITY TO ACHIEVE YOUR FULL POTENTIAL

Not at all ____________________________ A great deal

4. LOSS OF BOWEL CONTROL

Not at all ____________________________ A great deal

5. DEVELOPING CANCER

Not at all ____________________________ A great deal

6. DYING EARLY

Not at all ______________________________ A great deal

7. BEING A BURDEN (OR DEPENDING) ON OTHERS

Not at all ______________________________ A great deal

8. YOUR ATTRACTIVENESS

Not at all ______________________________ A great deal

9. FEELING ALONE

Not at all ______________________________ A great deal

10. FEELING OUT OF CONTROL

Not at all ______________________________ A great deal

11. FEELING "DIRTY" OR "SMELLY"

Not at all ______________________________ A great deal

12. YOUR ABILITY TO PERFORM SEXUALLY

Not at all ______________________________ A great deal

13. YOUR ABILITY TO HAVE CHILDREN

Not at all ______________________________ A great deal

14. PASSING THE DISEASE ONTO OTHERS

Not at all ______________________________ A great deal

15. BEING TREATED AS DIFFERENT

Not at all ______________________________ A great deal

16. HAVING SURGERY

Not at all ______________________________ A great deal

17. HAVING AN OSTOMY BAG

Not at all ______________________________ A great deal

18. PRODUCING UNPLEASANT ODORS

Not at all ______________________________ A great deal

19. YOUR ENERGY LEVEL

Not at all ______________________________ A great deal

20. YOUR FEELINGS ABOUT YOUR BODY

Not at all ______________________________ A great deal

21. INTIMACY

Not at all ______________________________ A great deal

22. LOSS OF SEXUAL DRIVE

Not at all ______________________________ A great deal

23. HAVING ACCESS TO QUALITY MEDICAL CARE

Not at all ______________________________ A great deal

24. THE UNCERTAIN NATURE OF YOUR DISEASE

Not at all ______________________________ A great deal

25. EFFECTS OF MEDICATION

Not at all ______________________________ A great deal

26. I am also concerned about:

____

____

____

Short Quality of Life Questionnaire for Inflammatory Bowel Disease

Name ____ Date____________

This questionnaire is designed to find out how you have been feeling during the last 2 weeks. You will be asked about symptoms you have been having as a result of your inflammatory bowel disease, the way you have been feeling in general, and how your mood has been. Please circle the number of your choice below each question.

1. How often has the feeling of fatigue or being tired and worn out been a problem for you during the past 2 weeks?
   1. All of the time
   2. Most of the time
   3. A good bit of the time
   4. Some of the time
   5. A little of the time
   6. Hardly any of the time
   7. None of the time

2. How often during the last 2 weeks have you delayed or canceled a social

Irvine, E. J., et. al. The short inflammatory bowel disease questionnaire: A quality of life instrument for community physicians managing inflammatory bowel disease. Am J Gastroenterology. 1996; 91(8):1571-8 Aug.

engagement because of your bowel problem?

1. All of the time
2. Most of the time
3. A good bit of the time
4. Some of the time
5. A little of the time
6. Hardly any of the time
7. None of the time

3. As a result of your bowel problems, how much difficulty did you experience doing leisure or sports activities you would liked to have done during the past 2 weeks?

    1. A great deal of difficulty; activities made impossible
    2. A lot of difficulty
    3. A fair bit of difficulty
    4. Some difficulty
    5. A little difficulty
    6. Hardly any difficulty
    7. No difficulty; the bowel problem did not limit sports or leisure activities

4. How often during the past 2 weeks have you been troubled by pain in the abdomen?

    1. All of the time

2. Most of the time
3. A good bit of the time
4. Some of the time
5. A little of the time
6. Hardly any of the time
7. None of the time

5. How often during the past 2 weeks have you felt depressed or discouraged?
   1. All of the time
   2. Most of the time
   3. A good bit of the time
   4. Some of the time
   5. A little of the time
   6. Hardly any of the time
   7. None of the time

6. Overall, in the past 2 weeks, how much of a problem have you had with passing large amounts of gas?
   1. A major problem
   2. A big problem
   3. A significant problem
   4. Some problem

5. A little trouble
6. Hardly any trouble
7. No trouble

7. Overall, in the past 2 weeks, how much of a problem have you had maintaining or getting to the weight you would like to be?
    1. A major problem
    2. A big problem
    3. A significant problem
    4. Some problem
    5. A little trouble
    6. Hardly any trouble
    7. No trouble

8. How often during the past 2 weeks have you felt relaxed and free of tension?
    1. All of the time
    2. Most of the time
    3. A good bit of the time
    4. Some of the time
    5. A little of the time
    6. Hardly any of the time
    7. None of the time

9. How much of the time during the past 2 weeks have you been troubled by a feeling of having to go to the bathroom even though your bowels were empty?

   1. All of the time
   2. Most of the time
   3. A good bit of the time
   4. Some of the time
   5. A little of the time
   6. Hardly any of the time
   7. None of the time

10. How often during the past 2 weeks have you felt angry as a result of your bowel problem?

   1. All of the time
   2. Most of the time
   3. A good bit of the time
   4. Some of the time
   5. A little of the time
   6. Hardly any of the time
   7. None of the time

Work Productivity and Activity Impairment Questionnaire:

Crohn's disease (WPAI-CD)

The following questions ask about the effect of your Crohn's disease on your ability to work and perform regular activities. *Please fill in the blanks or circle a number, as indicated.*

1. Are you currently employed (working for pay)? _____NO _____YES

   If NO, check "NO" and skip to question 6.

The next questions are about the **past seven days**, not including today.

2. During the past seven days, how many hours did you miss from work because of problems associated with your Crohn's disease? *Include hours you missed on sick days, times you went in late, left early, etc., because of your Crohn's disease. Do not include time you missed to participate in this study.*

   _____HOURS

3. During the past seven days, how many hours did you miss from work because of any other reason, such as vacation, holidays, time off to participate in this study?

   _____HOURS

4. During the past seven days, how many hours did you actually work?

   _____HOURS *(If "0", skip to question 6.)*

5. During the past seven days, how much did your Crohn's disease affect your productivity <u>while you were working</u>?

Think about days you were limited in the amount or kind of work you could do, days you accomplished less than you would like, or days you could not do your work as carefully as usual. If Crohn's disease affected your work only a little, choose a low number. Choose a high number if Crohn's disease affected your work a great deal.

Crohn's disease had no effect on my work	0	1	2	3	4	5	6	7	8	9	10	Crohn's disease completely prevented me from working

CIRCLE A NUMBER

6. During the past seven days, how much did your Crohn's disease affect your ability to do your regular daily activities, other than work at a job?

By regular activities, we mean the usual activities you do, such as work around the house, shopping, childcare, exercising, studying, etc. Think about times you were limited in the amount or kind of activities you could do and times you accomplished less than you would like. If Crohn's disease affected your activities only a little, choose a low number. Choose a high number if Crohn's disease affected your activities a great deal.

Crohn's disease had no effect on my daily activities	0 1 2 3 4 5 6 7 8 9 10	Crohn's disease completely prevented me from doing my daily activities

CIRCLE A NUMBER

WPAI-CD (Canadian English)

Work Productivity and Activity Impairment Questionnaire:

Ulcerative Colitis V2.0 (WPAI-UC)

The following questions ask about the effect of your ulcerative colitis on your ability to work and perform regular activities. *Please fill in the blanks or circle a number, as indicated.*

1. Are you currently employed (working for pay)? _____ NO _____ YES

   If NO, check "NO" and skip to question 6.

The next questions are about the **past seven days**, not including today.

2. During the past seven days, how many hours did you miss from work because of problems associated with your ulcerative colitis? *Include hours you missed on sick days, times you went in late, left early, etc., because of your ulcerative colitis. Do not include time you missed to participate in this study.*

   _____HOURS

3. During the past seven days, how many hours did you miss from work because of any other reason, such as vacation, holidays, time off to participate in this study?

   _____HOURS

4. During the past seven days, how many hours did you actually work?

   _____HOURS *(If "0", skip to question 6.)*

5. During the past seven days, how much did your ulcerative colitis affect your productivity while you were working?

Think about days you were limited in the amount or kind of work you could do, days you accomplished less than you would like, or days you could not do your work as carefully as usual. If ulcerative colitis affected your work only a little, choose a low number. Choose a high number if ulcerative colitis affected your work a great deal.

Consider only how much ulcerative colitis affected productivity while you were working.

Ulcerative colitis had no effect on my work	0	1	2	3	4	5	6	7	8	9	10	Ulcerative colitis completely prevented me from working

CIRCLE A NUMBER

6. During the past seven days, how much did your ulcerative colitis affect your ability to do your regular daily activities, other than work at a job?

By regular activities, we mean the usual activities you do, such as work around the house, shopping, childcare, exercising, studying, etc. Think about times you were limited in the amount or kind of activities you could do and times you accomplished less than you would

like. If ulcerative colitis affected your activities only a little, choose a low number. Choose a high number if ulcerative colitis affected your activities a great deal.

Consider only how much ulcerative colitis affected your ability to do your regular daily activities, other than work at a job.

Ulcerative colitis had no effect on my daily activities	0	1	2	3	4	5	6	7	8	9	10	Ulcerative colitis completely prevented me from doing my daily activities

CIRCLE A NUMBER

WPAI:UC V2.0 (US English)

OCTAVO – Patient experience questions for baseline data collection

	Question	Answer options
**Topic 1 - Patient involvement in decision making about IBD treatment**		
1	I want to be involved in choosing my treatment	Likert scale, 5 points<br>totally disagree, somewhat disagree, neutral, somewhat agree, totally agree
2	I was involved in choosing my current treatment	Likert scale, 5 points<br>totally disagree, somewhat disagree, neutral, somewhat agree, totally agree
**Topic 2 – Treatment options**		
3	Apart from vedolizumab, which other treatment options were you offered?	Free text
4	If you were offered different treatment options, why did you choose vedolizumab?	Free text
5	I was given information on all the treatment options available	'Not applicable' option and Likert scale, 5 points<br>totally disagree to totally agree
6	I received enough information on different treatment options to help me to choose the best treatment for me	'Not applicable' option and Likert scale, 5 points<br>totally disagree to totally agree
**Topic 3 - Patient values**		
7	Which of the following factors is **most important** to you	Select one answer only

	when choosing a treatment for IBD:<br>• Quick improvement of symptoms<br>• Managing symptoms in the long term<br>• Receiving treatment at home<br>• Risk of (serious) side effects	
8	When choosing a treatment for IBD, quick improvement of symptoms is important to me	Likert scale, 5 points<br>totally disagree, somewhat disagree, neutral, somewhat agree, totally agree
9	When choosing a treatment for IBD, long term management of symptoms is important to me	Likert scale, 5 points<br>totally disagree, somewhat disagree, neutral, somewhat agree, totally agree
10	When choosing a treatment for IBD, receiving treatment at home rather than in hospital is important to me	Likert scale, 5 points<br>totally disagree, somewhat disagree, neutral, somewhat agree, totally agree
11	When choosing a treatment for IBD, risk of (serious) side effects is important to me	Likert scale, 5 points<br>totally disagree, somewhat disagree, neutral, somewhat agree, totally agree
12	Tell us about anything else that is important to you when choosing a treatment for IBD	Free text
13	Tell us about any worries you have about vedolizumab treatment	Free text
