# Supplementary material for: Health-related quality of life outcomes among vedolizumab-treated patients with inflammatory bowel disease in the UK and Ireland: a 52-week observational study (OCTAVO)
Source: J Patient Rep Outcomes. 2025 Jul 1;9:80. doi: 10.1186/s41687-025-00846-9 (PMC12214154; doi:10.1186/s41687-025-00846-9)
Supplement: Supplementary file 2 — Supplementary Material 2 [file 41687_2025_846_MOESM2_ESM.docx]

**Supplementary Tables**

**Supplementary Table S1**: SIBDQ sub-scores^a^ at baseline and week 14, 26, and 52 for patients with CD and UC

|  | **CD** | | | | **UC** | | | |
| --- | --- | --- | --- | --- | --- | --- | --- | --- |
|  | Baseline | Week 14 | Week 26 | Week 52 | Baseline | Week 14 | Week 26 | Week 52 |
| N | 22 | 18 | 18 | 14 | 38 | 33 | 32 | 29 |
| **Bowel systems** | | | | | | | | |
| Mean (SD) | 3.8 (1.5) | 4.8 (1.3) | 4.2 (1.5) | 4.7 (1.1) | 3.9 (1.6) | 4.8 (1.6) | 5.0 (1.5) | 5.2 (1.6) |
| **Emotional health** | | | | | | | | |
| Mean (SD) | 3.5 (1.5) | 4.6 (1.4) | 3.9 (1.4) | 4.8 (1.1) | 3.9 (1.5) | 4.7 (1.7) | 4.9 (1.5) | 5.0 (1.7) |
| **Systemic symptoms** | | | | | | | | |
| Mean (SD) | 3.5 (1.4) | 4.4 (1.2) | 3.8 (1.3) | 4.9 (1.4) | 4.0 (1.6) | 4.5 (1.8) | 4.5 (1.4) | 4.6 (1.5) |
| **Social function** | | | | | | | | |
| Mean (SD) | 4.0 (2.1) | 4.9 (1.3) | 4.4 (1.7) | 5.5 (1.5) | 4.3 (1.8) | 5.0 (1.9) | 5.4 (1.5) | 5.6 (1.6) |

^a^Possible scores range from 1 to 7. Higher scores indicate better functioning

CD, Crohn’s disease; SD, standard deviation; SIBDQ, Short Inflammatory Bowel Disease Questionnaire; UC, ulcerative colitis

**Supplementary Table S2**: RFIPC sub-scores^a^ at baseline and week 14, 26, and 52 for patients with CD and UC

|  | **CD** | | | | **UC** | | | |
| --- | --- | --- | --- | --- | --- | --- | --- | --- |
|  | Baseline | Week 14 | Week 26 | Week 52 | Baseline | Week 14 | Week 26 | Week 52 |
| N | 22 | 18 | 18 | 14 | 38 | 33 | 32 | 29 |
| **Impact of disease** | | | | | | | | |
| Mean (SD) | 64.5 (20.5) | 48.6 (23.1) | 55.3 (23.2) | 48.2 (26.9) | 64.8 (20.4) | 48.8 (27.1) | 46.0 (26.1) | 46.1 (29.2) |
| **Sexual intimacy** | | | | | | | | |
| Mean (SD) | 48.9 (35.5) | 37.0 (33.1) | 40.9 (34.2) | 43.5 (36.2) | 51.4 (33.6) | 45.9 (36.3) | 42.5 (31.1) | 38.8 (35.4) |
| **Complication of disease** | | | | | | | | |
| Mean (SD) | 64.0 (27.2) | 54.4 (27.2) | 57.8 (25.5) | 55.5 (26.3) | 66.3 (28.7) | 54.5 (32.0) | 50.0 (27.9) | 51.9 (25.1) |
| **Body stigma** | | | | | | | | |
| Mean (SD) | 32.0 (21.1) | 23.1 (24.0) | 28.4 (22.8) | 20.2 (22.1) | 35.5 (24.4) | 25.8 (26.6) | 26.4 (26.2) | 26.6 (28.1) |

^a^Sub-scores range from 0 (least possible concern) to 100 (highest possible concern)

CD, Crohn’s disease; RFIPC, Rating Form of Inflammatory Bowel Disease Patient Concerns; SD, standard deviation; UC, ulcerative colitis
